# A Wavelet Evaluation of Some Leading Business Cycle Indicators for the German Economy

**DOI:** 10.1007/s41549-021-00060-8

**Published:** 2021-10-19

**Authors:** Jens J. Krüger

**Affiliations:** Technical University of Darmstadt, Department of Law and Economics, Hochschulstr. 1, Darmstadt, D-64289 Germany

**Keywords:** Business cycle forecasting, Leading indicators, Wavelet analysis, Phase difference, C49, E32, E37

## Abstract

Leading indicators are important variables in business cycle forecasting. We use wavelet analysis to investigate the lead-lag stability of German leading indicators in time-frequency space. This method permits a time-varying relation of the leading indicators to the reference cycle allowing simultaneously to focus on lead-lag stability at the specific business cycle frequencies. In this way we analyze an index of new orders, a survey-based index of business expectations, an index of stock market returns and the interest rate term spread. We confirm that most of these indicators are indeed leading the reference cycle most of the time, but the number of months leading varies considerably over time and is associated with a great deal of estimation uncertainty.

## Introduction

Leading business cycle indicators have traditionally received a great deal of attention in the macroeconomic forecasting community. The literature on both single leading indicators and composite leading indicators is substantial (see, among many others, Diebold and Rudebusch (1989, 1991), Emerson and Hendry (1996), Garnitz et al. (2019), Lahiri and Moore (1991), Oppenländer and Poser (1984), Stock and Watson (1989). Marcellino (2006) provides a comprehensive survey. Especially concerned with German experience are Bandholz and Funke (2003), Entorf (1993), Kholodilin and Siliverstovs (2006) and Lehmann (2020), inter alia.

The main purpose of leading indicators is short-term business cycle forecasting and identifying the turning points. Marcellino (2006, p. 885) defines six desirable properties of leading indicators: (i) consistent timing, (ii) conformity to the general business cycle, (iii) economic significance, (iv) statistical reliability of data collection, (v) prompt availability without major revisions, (vi) smooth month to month changes. For our analysis we may add the availability of long time series. Central to the performance of leading indicators is their ability to lead the cycle and the variability of this lead over time.

In this paper we apply wavelet analysis to analyze the properties of popular leading indicators for the German economy. We intend a purely in-sample investigation of the properties of the leading indicators. Applying wavelet analysis requires long time series at a monthly frequency which are only available for a limited set of leading indicators. From the results we can infer the cyclical properties of the leading indicators and their relations to the reference cycle. The results also allow to determine the lead (or lag) of the indicators and to investigate the stability of this lead (or lag) over time.

Wavelet analysis is a refinement of traditional frequency domain (spectral) analysis.^1^ It appears to be particularly adapted to the analysis of lead-lag relationships among variables at a specific band of frequencies for at least three reasons. First, wavelets allow for the analysis of relationships differentiated across time and frequencies in a unified methodological framework. Second, wavelets allow to focus on a particular band of frequencies which is particularly relevant for the application at hand (which, in our case, pertain to the frequencies relevant for business cycle analysis). Third, no parameters have to be estimated which implies that there is no need to be concerned about parameter stability over the sample period or the application of time-varying or regime-switching models.

The remainder of this paper is organized as follows. We start by introducing the toolbox of wavelet analysis provided by the continuous wavelet transform in Sect. 2. In Sect. 3 the data of the reference cycle and the four leading indicators which are in the focus of this paper are discussed. The univariate wavelet results for these data are discussed in Sect. 4, followed by the bivariate relations of the leading indicators to the reference cycle in Sect. 5. Section 6 concludes. An appendix provides an example with artificially generated data.

## Wavelet Analysis and Cross Wavelet Analysis

In econometrics, traditional time series analysis is done in the time domain by tracking the expectation of a variable over time, conditional on its own past and the past of other variables. Cross-correlation analysis in the time domain does not allow for focusing on fluctuations within specific frequency bands but instead relies on a mixture of all frequencies simultaneously. Less prevalent in econometrics (but much more in the natural sciences) is the application of spectral analysis in the frequency domain. By the Fourier transform, spectral analysis generates a representation of the time series by a weighted combination of sine or cosine waves at different frequencies. Here, the weights represent the importance of the fluctuations of a certain frequency to the overall variation of the time series. These weights are presumed to be constant over the whole time span under investigation. Thus, spectral analysis only provides resolution with respect to frequency but not over time.

Wavelet analysis can be viewed as a local form of frequency domain analysis. By the wavelet transform, we can investigate how the spectral characteristics of time series change over time and therefore gain both time and frequency resolution. A succinct characterization of wavelet analysis is providing a “spectral analysis as a function of time” (Aguiar-Conraria and Soares 2014, p. 364). Wavelet analysis is also advantageous compared to so-called windowed Fourier transform which means repeating the spectral analysis for a rolling window of time periods, each containing a subset of the entire time series. For this kind of analysis, the choice of window length is critical and is usually taken to be the same for all frequencies which may result in a suboptimal solution to the trade-off of time and frequency resolution (Rua 2012).

The toolbox provided by wavelet analysis is not only widely applied in the natural sciences, it also has a wide range of applications in economics and finance. Economic applications comprise the synchronization of economic activity and business cycles across European countries (Aguiar-Conraria and Soares 2011a; Rua 2010) and the co-movement of international stock markets (Rua and Nunes 2009). The relations of economic activity to other economic variables like stock market returns (Gallegati 2014a), the oil price (Aguiar-Conraria and Soares 2011b) or variables representing monetary policy (Aguiar-Conraria et al. 2008; Caraiani 2012) have also been investigated by wavelet methods. Concerning leading indicators the discrete wavelet transform (DWT) has been applied to improve the properties of composite leading indicators (Gallegati 2014b). Rua (2017) is another application to improve forecasting. See also Ramsey (2002) and Crowley (2007) for early surveys of the application of the discrete wavelet transform to decompose single time series, also known as multi-resolution analysis. Percival and Walden (2000) provide a book-length mathematical treatment. Many forecasting studies rely on the discrete wavelet transform to decompose a time series into different scales and then to try to forecast the parts more exactly (see Fernandez 2008; Bruzda 2020 and the references cited therein).

In this work we apply the continuous wavelet transform (CWT) which is advocated by Aguiar-Conraria and Soares (2014) for discovering patterns or hidden information. The following formal description of wavelet analysis and the concepts used in the empirical application borrows from several sources, mainly (Aguiar-Conraria and Soares 2014; Rua 2012; Torrence and Compo 1998) and the tutorial of Rösch and Schmidbauer (2018). In the latter, the software used, namely the R-package “WaveletComp” (version 1.1), is thoroughly described. Where certain normalizations are introduced we follow the definitions in Rösch and Schmidbauer (2018).

For a single time series $\{y_{t}\}$ in discrete time with $t=1,...,T$ the basic wavelet is a function of the position in the time domain $\tau$ and the position in the frequency domain *s* (expressed inversely by the scale or period). It is defined as

$$\begin{aligned} W_{y}(\tau ,s)={\displaystyle \sum _{t=1}^{T}}y_{t}\dfrac{1}{\sqrt{s}}\psi ^{*}\left( \dfrac{t-\tau }{s}\right) \end{aligned}$$

with $\psi ^{*}(\cdot )$ as the complex conjugate of the Morlet mother wavelet $\psi (t)=\pi ^{-1/4}e^{i\omega t}e^{-t^{2}/2}$ (with imaginary unit $i=\sqrt{-1}$) which is shifted in time by $\tau$ and scaled by *s* (translation $\tau$, dilation *s*).^2^ The angular frequency $\omega$ is usually set to $\omega =6$. Among the wavelet forms discussed in the literature the Morlet wavelet is most frequently used since it is viewed as providing the best compromise of accuracy in both time domain and frequency domain (see Aguiar-Conraria and Soares 2014, p. 352). It is also implemented in the WaveComp package.

Concepts derived from the basic wavelet are the amplitude $A_{y}(\tau ,s)=\dfrac{1}{\sqrt{s}}|W_{y}(\tau ,s)|$ and the wavelet power spectrum

$$\begin{aligned} P_{y}(\tau ,s)=\dfrac{1}{s}|W_{y}(\tau ,s)|^{2} \end{aligned}$$

as its square. The magnitude of the wavelet power spectrum reveals the importance of periodic fluctuations of scale (period) *s* at time $\tau$. Averaging the wavelet power spectrum along the time dimension $\tau$ gives the average power spectrum with the same interpretation as the power spectrum of traditional spectral analysis.

A further derivative concept of the basic wavelet is the wavelet phase angle

$$\begin{aligned} \phi _{y}(\tau ,s)=\arctan \left( \dfrac{\mathrm {Im}(W_{y}(\tau ,s))}{\mathrm {Re}(W_{y}(\tau ,s))}\right) \end{aligned}$$

where $\mathrm {Re}(\cdot )$ and $\mathrm {Im}(\cdot )$ denote the real and imaginary parts, respectively. The wavelet phase angle $\phi _{y}(\tau ,s)\in [-\pi ,\pi ]$ represents the shift of periodic fluctuations relative to the origin $\tau$ in the time domain in angular form. We return to the concept of the phase angle when we next discuss the bivariate wavelet transform.

With two time series $\{x_{t}\}$ and $\{y_{t}\}$ for $t=1,...,T$ we are able to compute two univariate wavelets $W_{x}(\tau ,s)$ and $W_{y}(\tau ,s)$. In addition we can compute the cross wavelet transform (XWT)

$$\begin{aligned} W_{xy}(\tau ,s)=\dfrac{1}{s}W_{x}(\tau ,s)W_{y}^{*}(\tau ,s) \end{aligned}$$

from the two wavelets with the asterisk denoting complex conjugation. The cross wavelet power spectrum is then computed by $P_{xy}(\tau ,s)=|W_{xy}(\tau ,s)|$. While the univariate wavelet power spectra represent the variation of the respective time series, the cross wavelet power spectrum represents the covariation.

Based on this, wavelet coherence is computed as

$$\begin{aligned} \rho _{xy}(\tau ,s)=\dfrac{|S(W_{xy}(\tau ,s))|^{2}}{S(P_{x}(\tau ,s))\cdot S(P_{y}(\tau ,s))} \end{aligned}$$

and is bounded by $0\le \rho _{xy}(\tau ,s)\le 1$. It can be viewed as the squared local correlation in time-frequency space. Herein, $S(\cdot )$ symbolizes the smoothing operation across time and scale (see e.g. Grinsted et al. 2004 or Cazelles et al. 2007 for details). This smoothing is necessary since otherwise coherence would be identically one for all $\tau$ and *s* by construction.

In the WaveletComp package the wavelets are computed by fast Fourier transform algorithms (see Torrence and Compo 1998 for some details). In addition, the wavelets are corrected for bias according to Liu et al. (2007) for the univariate case and according to Veleda et al. (2012) for the bivariate case.

The significance of the coherence values against the benchmark of no common periodicity is usually established by simulation. Simulated are independent normal draws of the two time series, i.e. two independent processes with the same empirical spectrum as the data (so-called Fourier randomization). All simulations are based on 1000 repetitions. In the plots of the wavelet power spectrum and the wavelet coherence black contour lines are added which enclose the areas of significance on a 10 percent level.^3^

Of central importance for the present application is the wavelet phase difference determining the lead-lag relation of the leading indicators to the reference cycle and investigating its stability over time. The phase difference is defined as

$$\begin{aligned} \phi _{xy}(\tau ,s)=\arctan \left( \dfrac{\mathrm {Im}(W_{xy}(\tau ,s))}{\mathrm {Re}(W_{xy}(\tau ,s))}\right) \end{aligned}$$

and is equal to the phase difference in angular form $\phi _{x}(\tau ,s)-\phi _{y}(\tau ,s)$. If the phase difference is equal to zero then both time series are moving together (i.e. have the same phase angle). Abbreviating $\phi _{xy}\equiv\phi _{xy}(\tau ,s)$ it is said that both time series are in phase with $x_{t}$ leading $y_{t}$ if $\phi _{xy}\in (0,\frac{\pi }{2})$. If $\phi _{xy}\in (-\frac{\pi }{2},0)$ the series are in phase with $y_{t}$ leading $x_{t}$, if $\phi _{xy}\in (\frac{\pi }{2},\pi )$ the series are out of phase (or anti-phase) with $y_{t}$ leading $x_{t}$ and if $\phi _{xy}\in (-\pi ,-\frac{\pi }{2})$ the series are also out of phase now with $x_{t}$ leading $y_{t}$ (see Rösch and Schmidbauer 2018, p. 7 for a graphical illustration).

The phase difference in angular form $\phi _{xy}$ can be converted into real time units (i.e. months) by dividing by the angular frequency corresponding to scale *s*. This means computing $\varDelta t=\phi _{xy}\cdot s/(2\pi f)$ where *f* is the underlying frequency of the data (i.e. $f=1/12$ in the case of monthly data) and *s* is the chosen scale or the average over a range of scales. A main advantage of applying the wavelet analysis is that the assessment of the lead-lag structure and the investigation of its stability can be focused on the relevant scales (which are the scales related to business cycle frequencies in our application).

For the interpretation of the phase difference it is important to focus on regions in the time-scale space inside the so-called cone of influence (COI) and where coherence is high.^4^ As stressed by Funashima (2017), the phase difference is not interpreted in a consistent way in the literature. To be totally clear about this critical issue, the appendix provides an example based on simulated data. This appendix also explains the structure of the figures used to present the empirical results below.

According to Ge (2008) there are no good statistical tests for the phase difference available. We attempt to assess the precision with which the phase difference is estimated by using blocks bootstrapping to compute confidence intervals for the median phase difference. This requires recomputing the XWT for 1000 bootstrap samples generated by resampling over blocks with a fixed length of 24 months and recomputing the phase shift in time units for each resample (using the function tsboot() in the “boot” package for R). The 0.05 and 0.95 quantiles then constitute the 90 percent confidence intervals.

## Time Series Data

The subsequent analysis of the lead-lag relationships is based on the German production index for manufacturing as the time series used for representing the reference cycle. Four examples of popular leading indicators are examined: an index of new orders in manufacturing, a survey-based index of business cycle expectations, the stock market performance represented by the returns of a major stock market index and the spread of a long-term and a short-term interest rate. The first two indicators are directly related to aspects of the real economy while the latter two are financial indicators. All these leading indicators are readily available from public data sources and are utilized by decision makers presumably without further data processing.^5^ The data are mostly taken from the time series database of the German central bank (Deutsche Bundesbank) to be found at https://www.bundesbank.de/en/statistics/time-series-databases. For all these leading indicators the data are available for a long time span at monthly frequency.

The production index is the index of output in the production sector (i.e. manufacturing) excluding construction, calendar and seasonally adjusted,^6^ which is available for the period 1950:01-2021:03 and normalized to 2015 = 100 (meaning that the mean of the months in 2015 is equal to 100). This index is provided in real units at constant prices. In the time series database of the German central bank this series has the code BBDE1.M.DE.Y.BAA1.A2P100000.G.C.I15.L. We use this index as the reference cycle since it is available for a long time period at a monthly basis and because manufacturing is more responsive to changes in overall economic activity than services. By contrast, GDP would be a more comprehensive measure which is, however, only available at quarterly frequency.

An obvious leading indicator naturally preceding production is the index of new orders, i.e. the index of new orders received in manufacturing (at constant prices, calendar and seasonally adjusted, period 1952:01-2021:04, 2015=100, in the database listed with code BBDE1.M.DE.Y.AEA1.A2P300000.F.C.I15.L).

Business cycle expectations are represented using data from the business outlook of the ifo Business Climate Index. This index originates from a monthly survey conducted by the ifo Institute for Economic Research in Munich, Germany, asking several thousand firms in manufacturing, construction, wholesaling and retailing about the current business situation (in terms of good | satisfactory | poor) and their expectations for the next six months (in terms of more favorable | unchanged | less favorable) with the responses aggregated to index numbers. Methodological details are provided by Ruppert (2007) as well as Sauer and Wohlrabe (2020). Lehmann (2020) also provides more detail about the construction of the indicators and a recent thorough review of studies of the forecasting properties.

We use the balances (i.e. the difference of the share of favorable and the share of less favorable answers) of business cycle expectations in manufacturing from the website https://www.ifo.de/en/umfragen/time-series. These are available in seasonally adjusted form during 1991:01-2021:07 for entire Germany. We extend the series backwards by the “Historical Time Series for Western Germany (1960–1990)” to 1960:01 (see Sommer and Wohlrabe 2016 and Sauer 2020). Since the more recent part of the time series is for reunified Germany and is seasonally adjusted whereas the earlier part is for western Germany and is not seasonally adjusted we apply annual differencing over 12 months.^7^ The problems from splicing the two parts together are also attenuated by the local nature of wavelet analysis. No break or anomalous behavior around 1990/91 is visible in the wavelet plots analyzed below.

In addition, we construct an alternative expectations index by splicing together an older series from 1969:01-2006:01 (base 2005=100) and a more recent series 2005:01-2021:07 (base 2015=100) of the business cycle expectations index. The more recent series is re-based to 2005=100 and extends the older series starting with 2005:01. The final series is then re-based to 2015=100. We find a close agreement of both parts during the overlapping months. Taking annual differences of this series, we find a high correlation ($\rho \approx 0.96)$ with the differenced balances described in the previous paragraph. Both series agree very closely when brought to a common scale by standardizing and plotted over each other.

In addition, we also explore two financial variables, i.e. stock market returns and interest rate spreads, as leading indicators. Stock and Watson (2003a) give a thorough discussion of the predictive power of financial variables in business cycle research. Key advantages of these financial variables are that they are readily available, observed with negligible measurement error and not subject to data revisions. The stock market performance is a potentially leading indicator since the stock market quickly reacts to news which are related to changes in overall economic activity and therefore affect expected future firm profitability. The theoretical justification for interest rate spreads as leading indicators originates from the fact that monetary policy can influence short-term interest rates more easily than long-term interest rates. Tighter monetary policy then lets short-term interest rates rise while the long-term rates react much less. This reduces the spread (the yield curve flattens) and may be associated with lower output growth in the future. See Adrian and Estrella (2008); Estrella et al. (2006); Estrella and Mishkin (1998) for a more detailed expositions of the argument.

The stock market development is represented by the DAX performance index, again taken from the time series database of the German central bank (end of month, period 1959:12-2021:06, 1987:12=1000, code WU3141). The interest term spread is the difference of a long-term and a short-term interest rate. As long-term interest rate we use the average yields on debt securities outstanding issued by residents with mean residual maturity of more than 9 and up to 10 years which is available for the period 1973:04-2021:06 in the time series database of the German central bank (code BBSIS.M.I.UMR.RD.EUR.A.B.A.R0910.R.A.A._Z._Z.A). The short-term interest rate series is a three-month interest rate spliced together from the monthly average money market rates reported by Frankfurt banks for three-month funds rate for the period 1959:12-1998:12 and the monthly average EURIBOR three-month funds during the period 1999:01-2021:06 (with codes SU0107 and SU0316, respectively).

We apply specific transformations of the series to eliminate the trend. The time series of the production index is converted to the annual percentage growth rate (by $100\cdot \Delta _{12}\ln y_{t}=100\cdot (\ln y_{t}-\ln y_{t-12})$).^8^ The new orders index is transformed in the same way whereas the expectations index is transformed by $\Delta _{12}y_{t}$ as explained above. The DAX performance index is converted to annual returns, analogous to the annual growth rate. No transformation is applied to the term spread since any differencing would exaggerate the importance of high-frequency fluctuations and distort the lead-lag analysis at the business cycle frequencies. When we refer to the index series in the following we actually mean the index series that have been subjected to the respective transformations.

**Fig. 1:**
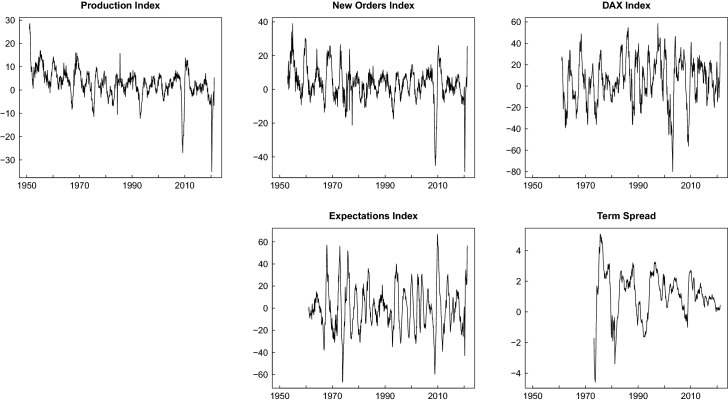
Time series plots production index and leading indicators

The time series resulting from the transformations are depicted in Fig. 1. Clearly the transformations successfully removed trends while preserving the relevant fluctuations at the business cycle frequencies. The upper-left panel shows the annual growth rates of the production index with clearly visible cyclical variations. The asymmetry of the business cycle (Morley and Piger 2012) with sharp and short recessions and booms lasting for longer periods is also visible. Major recessions occurred in the mid of the 1970s and the beginning of the 1980s and 1990s. The impact of the Great Recession and the recent COVID-19 pandemic is striking.

These characteristics are also visible to some extent in the time series plots of the four leading indicators which are depicted in the other panels of the figure. The new orders index appears equally volatile as the production index with downward peaks in the vicinity of the recessions, exceptionally strong in the case of the Great Recession and again the COVID-19 crisis. The recessions are also visible in the case of the expectations index, but there are also more spurious identifications in this case. The annual DAX returns are also quite volatile with several upward and downward peaks. The largest downturn follows the collapse of the dotcom bubble at the beginning of the 2000s where just a small business cycle downturn can be observed. The second largest downturn is associated with the Great Recession. From the visual inspection it is less clear how these stock market fluctuations are related to business cycle fluctuations. The term spread is usually positive (i.e. the long-term rate is larger than the short-term rate) but tends to turn into the range of negative values in the vicinity of recessions. These downward peaks were particularly strong in the 1970s and the beginning of the 1980s but have been considerably moderated thereafter. From the plots of the financial indicators the impact of the COVID-19 crisis is not visible at all.

**Fig. 2:**
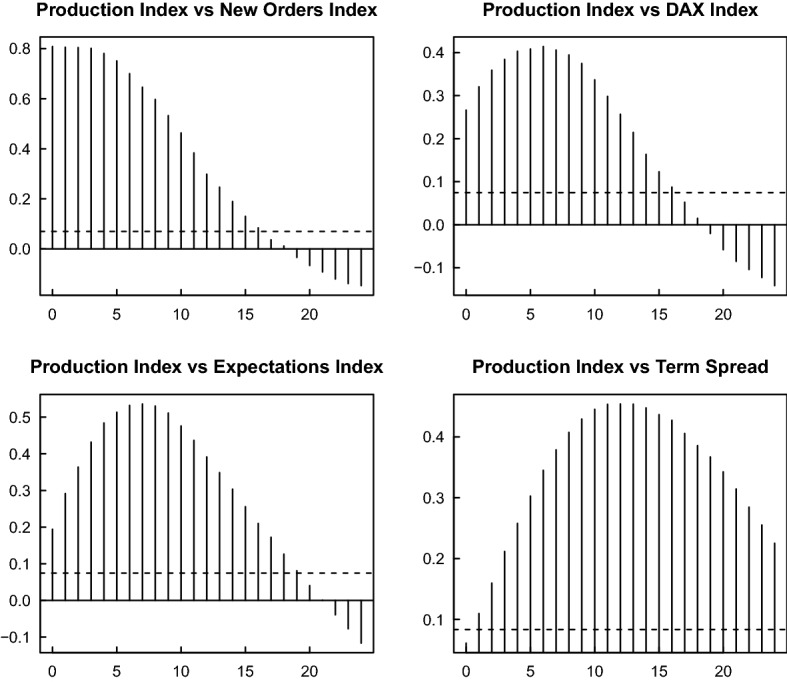
Cross correlation of the production index and leading indicators

To gain a first impression of the lead properties in the time domain we show the cross correlations of the leading indicators with the production index in Fig. 2. The lead periods with the maximum cross correlations are about 3 months for the new orders index and roughly 7 months for the expectations index. For the financial indicators we find about 6 months for the DAX returns and about 12 months for the interest rate spread, with lower maximum cross correlations, however. These cross correlations represent averages over the entire sample period and are not focused on a particular band of periods (i.e. the periods relevant for business cycle phenomena). This leads us to the following frequency domain analysis by wavelets which is also operating locally on the time scale.

## Univariate Wavelet and Power Spectra

In this section we show the univariate wavelet estimates and the average power spectra for a more detailed account of the cyclical properties of the time series. An illustrative example in a controlled simulation environment is discussed in the appendix.

**Fig. 3:**
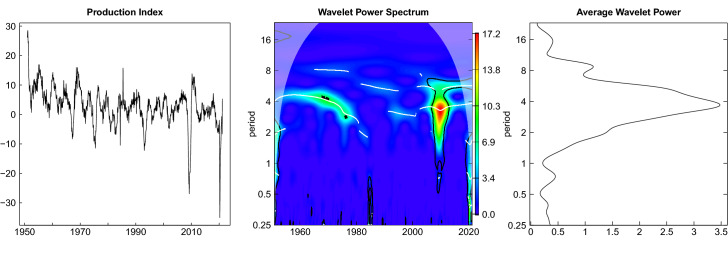
Wavelet power spectrum production index

**Fig. 4:**
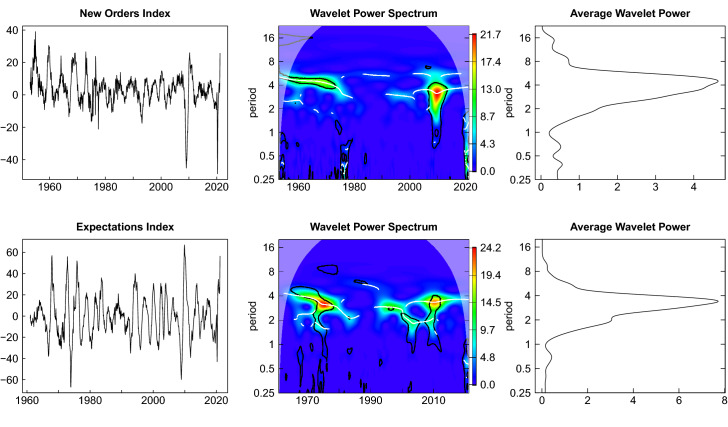
Wavelet power spectrum—new orders and expectations index

**Fig. 5:**
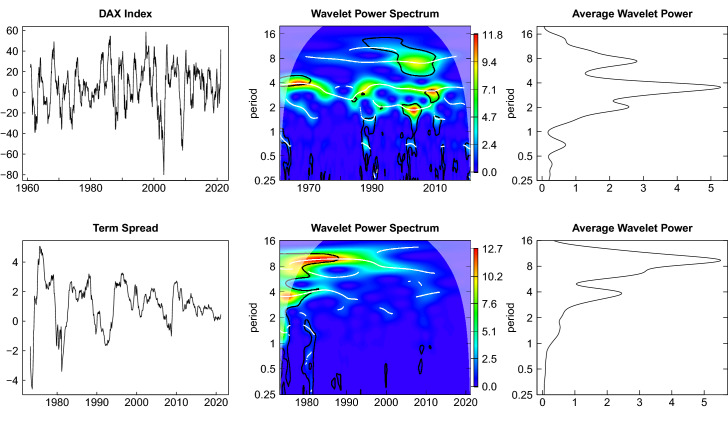
Wavelet power spectrum DAX index and interest rate spread

In Figs. 3, 4 and 5 the univariate wavelet plots are shown in the middle panel with the corresponding time series plots repeated in the left panel and the average wavelet power spectrum depicted in the right panel. High values of wavelet power are colored in red and yellow while lower values are depicted in green and blue color. Figure 3 shows the estimated wavelet power spectrum of the production index with ridges (indicated by the white lines) at the relevant business cycle periodicities of 6–32 quarters corresponding to 1.5–8 years as shown on the ordinate on a log scale.^9^ The black lines show that these values are significantly different from white noise on a 10 percent level. Exceptionally large values of the wavelet power spectrum appear in the vicinity of the Great Recession. The average wavelet power confirms that fluctuations within a period band of roughly 2–8 years are dominating.^10^

The presentation of the leading indicators is split into two figures. Figure 4 shows the estimates for the new orders index and the expectations index. The wavelet power spectrum and the average wavelet power of the new orders index look very similar to that of the production index. The fluctuations of the expectations index appear to be more regular within the range of business cycle periodicities for the entire sample period and the average wavelet spectrum is more concentrated in these periodicities. The impact of the Great Recession is less pronounced here.

The two financial market related leading indicators are depicted in Fig. 5. The fluctuations of the DAX index reveal multiple dominating scales within the range of business cycle periodicities. There is a substantial ridge at a period of about 4 years. This is also reflected in the multimodal shape of the average wavelet power spectrum for this series. In the case of the term spread there is a dominating ridge with a period of more than 8 years, but also a less pronounced ridge at a lower period of about 4 years. Both fluctuations vanish until the end of the time span under investigation, but also lead to the two peaks of the average wavelet power spectrum.

In sum, we see that all series have substantial variation in the range of periodicities which are of particular importance for business cycle analysis. These fluctuations are clearly visible from the average wavelet spectra. From the wavelet power spectra we can also infer that the importance of these fluctuations varies over time, however. Revealing those aspects is the value added of wavelet analysis over traditional spectral analysis. In the following section we look at the bivariate relations of the leading indicators with the production index by means of the wavelet coherence. This is associated with the assessment of the lead-lag relations using the phase difference.

## Wavelet Coherence and Lead-Lag Analysis

**Fig. 6:**
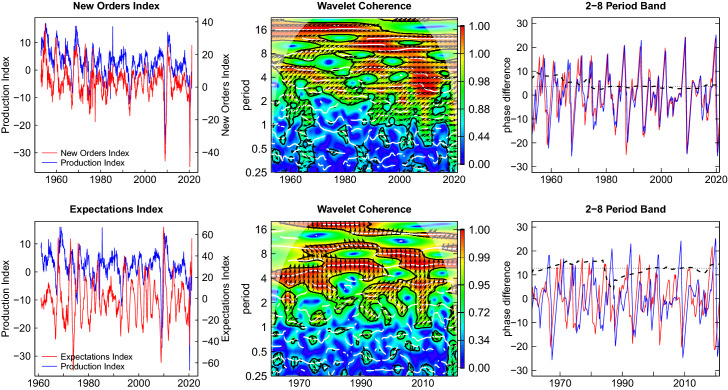
Wavelet coherence and phase difference new orders and expectations index

**Fig. 7:**
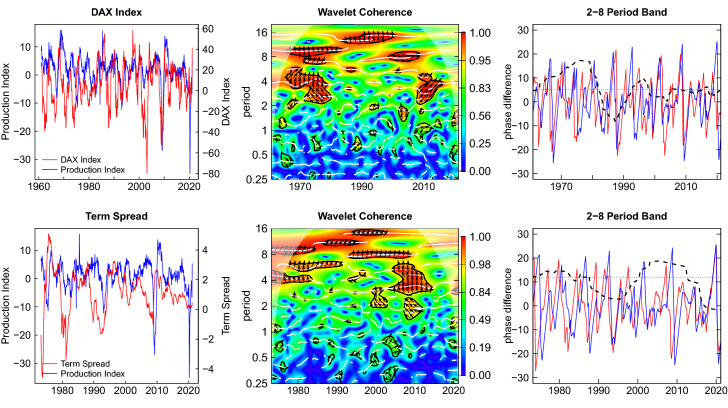
Wavelet coherence and phase difference—DAX index and interest rate spread

In the Figs. 6 and 7 the relations of the four leading indicators to the reference cycle are depicted. In the left panel, the time series of the respective leading indicator and the production index are plotted together. This gives a tentative impression of a lead-lag relation. The middle panel shows the wavelet coherence in time-period space. The maximum achievable coherence is equal to one, shown in red color. Lower coherence values are depicted in colder colors. For the 2-8 period band the right panel shows the phase angles (blue and red lines) and the phase difference as dashed black line. All angles are converted to time units, i.e. months, as shown on the ordinate. In addition, the median phase difference is shown as a dotted horizontal line. This type of plot is also explained for the simulation example in the appendix.

In all coherence plots we find extended areas of substantial coherence values in both time and period dimension. These areas are enclosed by black lines indicating that these coherences are significant on a 10 percent level. This holds for the business cycle periodicities with some interruptions but also for cycles with longer periods of about 16 years. In the case of the DAX index we find high coherence in the range of business cycle periodicities arise only around 1970 and 2010 and are more prevalent at higher periods. Around the Great Recession large coherence values can also be observed for lower periods at a value of 4 years, pointing to a deep impact which is not lasting for long. This phenomenon is also observed in other studies, e.g. Caraiani (2012). In the recent COVID-19 crisis a similar effect seems to emerge at the right end of the plots.

Within the regions of high coherence, the upward pointing arrows give an indication of a lead of the leading indicator. The horizontal arrows indicate the absence of a lead or lag. In Fig. 6 this is clearly visible in the case of the new orders index where the lead-lag relation to the reference cycle is attenuated in the 2000s around period 4. For the expectations we find strongly upward pointing arrows showing a pronounced lead with respect to the reference cycle. In the case of the financial leading indicators in Fig. 7 the areas of high coherence are also present but these appear to be less extended than in the case of the real leading indicators in Fig. 6.

The plots on the right show the phase difference at the business cycle frequencies averaged over a period of 2–8 years, deliberately excluding highest frequencies which can be particularly disturbing as the example in the appendix demonstrates.^11^ We observe that the dashed line representing the phase difference is rather stable for the new orders index and the expectations index, but varies widely in the case of the financial leading indicators. The leading indicators are indeed leading most of the time but the financial indicators are also occasionally lagging.^12^

The median lead times over the entire sample period are 3.6 months for new orders, 12.8 months for expectations, 5.1 months for the DAX index and 12.1 months for the interest spread. The corresponding 90 percent confidence intervals of the medians obtained by blocks bootstrapping are [2.801, 5.416], [12.341, 18.241], [0.719, 8.326] and [11.839, 23.292]. None of the intervals encloses the zero value. Nevertheless, the intervals are rather wide and show that substantial estimation uncertainty is involved.

The phase lead of the new orders is most stable and the confidence interval is narrow. The expectations also have a positive phase lead with is rather stable around a median level of about one year. It is puzzling that this value is much larger than the horizon for which the expectations are asked in the survey (6 months). This may suggest that the horizon which the survey respondents have in mind may be actually longer than the nominal horizon indicated by the question, but this is an open issue for future research.

The DAX index is leading in the median but the lead is unstable. The reason may be that the market not only depends on the business cycle expectations but also on a multitude of other causes for price chances (Stock and Watson 2003a). The median lead of the term spread of about a year is substantial, but also shows a great deal of variability. Forecasting exercises of U.S. recessions reveal similar lead times (see e.g. Estrella and Mishkin 1998; Kauppi and Saikkonen 2008). Interestingly, the term spread appears to loose its leading property in the most recent years, probably because of the sustained low interest policy enforced by the European Central Bank.

To summarize, we can extract the following core properties for practical business cycle analysis. The lead of the new orders is short (about one quarter) and is rather stable which lets new orders be a reliable short-run leading indicator. The expectations index has a longer lead (about one year in the median) and is also quite stable. In contrast, the DAX index is leading for one or two quarters but this lead time is rather variable. Finally, the term spread is associated with a widely varying lead time. The term spread looses its function as a signal in recent times, presumably as a cause of the low interest policy.

## Further Discussion and Conclusion

The bottom line obtained from the wavelet-based lead-lag analysis in this paper is that the four single leading indicators are indeed leading the reference cycle most of the time and to a considerable extent. However, it it also apparent that the duration of the lead is variable and is associated with substantial uncertainty. This assessment holds in particular for the financial indicators. The lead of the DAX index is short and rather variable. The lead of the interest term spread is positive throughout but varies widely around its median of about 12 months. For the real indicators we find that the lead of the new orders index is short but quite stable around its median. In this case, the median lead is relatively precisely estimated as can be inferred from the width of the confidence interval. Although subject to considerable estimation uncertainty, the lead of the expectations index appears to be closely associated with its median of about 12 months during the entire time span under investigation. In all cases the confidence intervals of the median lead not enclose zero.

Regarding specific episodes we observe that the recessions at the beginning of the 1990s and the 2000s are less pronounced in the German economy. These recessions have been considered to be different from previous recessions in the U.S. and dated to 1990–1991 and 2001 (see Marcellino 2006, pp. 949f. and Stock and Watson 2003b). In the wavelet power spectra of the reference cycle and the leading indicators the impact of these recessions appears to weaken the fluctuations at the relevant periodicities (with the exception of the DAX index). The smaller impact of these recessions in Germany may be attributed to special effects of the German reunification and the fact that the German economy was less affected by the market exit of dotcom firms. The stamp in the wavelet coherence plots is not clearly identifiable and an impact on the phase difference is only apparent for the financial leading indicators. Another relevant episode is the financial crisis 2007/08 followed by the Great Recession which is strikingly visible in the time series of the reference cycle. It also has an impact on the wavelet power spectra and large coherence values are extending to lower periods of about 2 years. This points to only a transitory effect of the Great Recession. The same characterization applies to the recent COVID-19 crisis but we are not observing the whole story so far. Despite that, the Great Recession exerts no visible effect on the phase difference lines and therefore appears not to have much impact on the lead-lag relations of the leading indicators to the reference cycle.

Future research may pay more attention to longer-run cycles which are visible in some of the wavelet power spectra and have been scrutinized as Kuznets cycles in earlier work (see Kuznets 1958 as well as Howrey 1968). Another research option would be to extend the evaluation to composite leading indicators (CLI) to see whether the broader information basis of these indicators pays off in terms of lead-lag stability. Paying more attention to out-of-sample forecast evaluation is a further research prospect, especially using the principal components weights to generate a CLI for a future period and to evaluate this CLI out of sample. In this context, the concept of targeted predictors from Bruzda (2020) may also be invoked and evaluated by the wavelet analysis tools. Nowcasting may also be a further application area, but is more in the domain of the discrete wavelet transform as already applied in Gallegati (2014b). Finally, cross-country linkages of CLIs could be analyzed in detail (i.e. by relating CLIs from other countries to the production index of a specific country under investigation).

## Appendix Example

To pick up the concerns of Funashima (2017) and to be totally clear about the correct interpretation of the phase difference as an angle and its transformation into a lead or lag expressed in terms of months we discuss a simple example with artificially generated data in this appendix. The setting of the data generating process is similar to Aguiar-Conraria and Soares (2014).

The series $y_{t}$ is generated as a periodic sine function with frequency 1/6 (period 6) and some noise $u_{t}$ added

$$\begin{aligned} y_{t}=3\cdot \sin \left( 2\pi \tfrac{1}{6}t\right) +u_{t}\text { for }t=\tfrac{1}{12},...,\tfrac{T}{12} \end{aligned}$$

with time proceeding in monthly steps and a total time series length of $T=600$ observations. This series can be understood as the reference cycle.

The series $x_{t}$ is generated similarly but is lagging behind $y_{t}$ by 10 units of time (months) during the first half of the sample and is leading $y_{t}$ by 5 months during the second half

$$\begin{aligned} x_{t}&=4\cdot \sin \left( 2\pi \tfrac{1}{6}\left( t-\tfrac{10}{12}\right) \right) +v_{t}\text { for }t=\tfrac{1}{12},...,\tfrac{T/2}{12}\\ x_{t}&=4\cdot \sin \left( 2\pi \tfrac{1}{6}\left( t+\tfrac{5}{12}\right) \right) +v_{t}\text { for }t=\tfrac{T/2+1}{12},...,\tfrac{T}{12}. \end{aligned}$$

This series may represent another variable which is related to the reference cycle, e.g. a leading indicator. For both series, the noise is generated as a sequence of iid normal random draws $u_{t}$ and $v_{t}$ with zero mean and variance equal to $0.5^{2}$. Note that the difference in the amplitudes of (3 for $y_{t}$ and 4 for $x_{t}$) is arbitrary and inessential for this analysis.

**Fig. 8:**
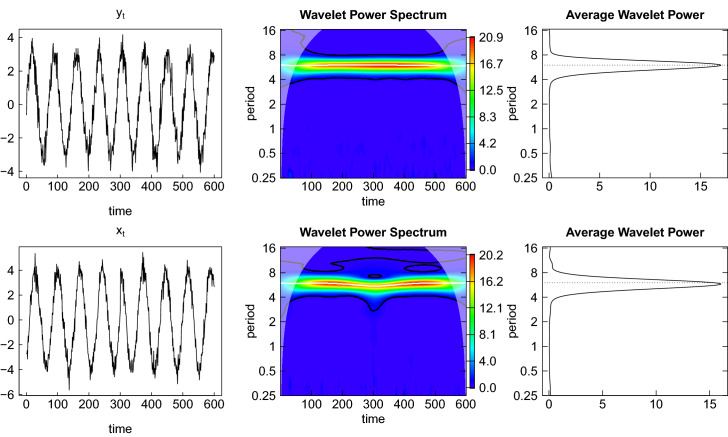
Time series and univariate wavelets

These aspects of the data generation are clearly visible in Fig. 8 showing the time series realizations of $y_{t}$ and $x_{t}$ on the left and the univariate wavelet power spectra in the middle. There is a narrow band of higher values (in yellow to red color) around period 6 (corresponding to the frequency 1/6) in the case of both variables. The cone of influence is also clearly visible. The white horizontal line represents the ridge of the largest wavelet power. This ridge is enclosed by black lines indicating the area of significant values on a 10 percent level (computed based on 1000 simulations). The dominance of the fluctuations at period 6 is also visible in the plots for the average wavelet power on the right side, which is an estimate of the power spectrum generated by averaging each period of the wavelet power spectrum along the time axis (depicted on a log scale). The peak there is exactly at period 6, as indicated by the dotted line.

**Fig. 9:**
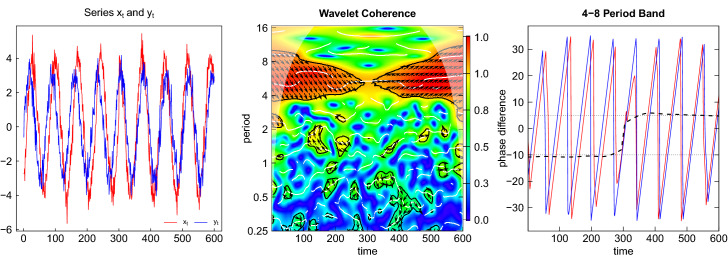
Wavelet coherence and phase difference

The left panel of Fig. 9 again shows the two time series $y_{t}$ and $x_{t}$ now plotted over each other. It is evident that the blue line for $y_{t}$ is leading during the first half of the sample, while the red line for $x_{t}$ is leading during the second half (by a smaller number of months). In the middle panel the wavelet coherence of $y_{t}$ and $x_{t}$ is plotted. The red shades around period 6 show a strong association of the two time series around the frequency 1/6. This association becomes weaker in the middle part of the sample where the lead-lag relationship changes. The areas enclosed by the black lines contain those coherence values which are significantly different from white noise on a 10 percent level. We observe that the coherence values in the middle part around time step 300 are not significant.

Within the significant regions the arrows indicate direction and strength of the lead-lag relationship. The downward pointing arrows in the left part of the coherency plot indicate that $y_{t}$ is leading $x_{t}$ (or $x_{t}$ is lagging $y_{t}$) while the upward pointing arrows in the right part indicate that $x_{t}$ is leading $y_{t}$ (or $y_{t}$ is lagging $x_{t}$). Notice that the arrows are more steeply pointing downwards than upwards, corresponding to the longer lead of $y_{t}$ (10 months) in the first half of the sample compared to the shorter lead of $x_{t}$ (5 months) in the second half.

The phase difference in time units is depicted in the right panel of the figure averaged over the relevant range of periods from 4 to 8 years. The red and blue lines are the phase angles of $x_{t}$ and $y_{t}$, respectively, and the horizontal black dashed line is the phase difference. The scale is converted into time units by dividing by the angular frequency corresponding to the average period or scale $\bar{s}$, i.e. $\varDelta t=\phi _{xy}\cdot \bar{s}/(2\pi f)$. We observe that the phase difference is close to the value $-10$ during the first half of the sample, indicating that $y_{t}$ is leading $x_{t}$ by 10 months. In the second half the line turns to the positive range and is close to a value of $+5$, now indicating that $x_{t}$ is leading $y_{t}$ by 5 months. This behavior exactly resembles the lead-lag relationship of the two variables as induced by the data generation.

**Fig. 10:**
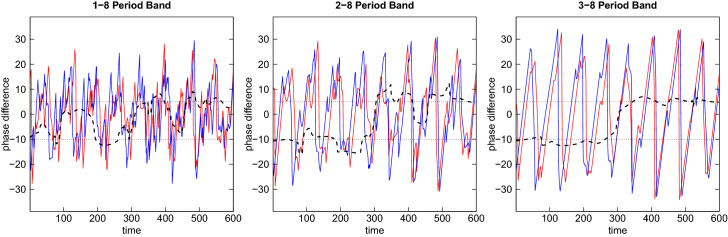
Phase difference for different period bands

It should be noted that the precision with which the phase difference is determined depends on the choice of the band of periods over which it is averaged. Figure 10 shows the phase difference for different period bands. The upper bound is fixed at 8 whereas the lower bound varies from 1 to 2 to 3. It is apparent that the fluctuations of the phase difference become much larger when the lower bound is smaller. This originates from the fact that with a smaller lower bound more fluctuations of a higher frequency are included and the source of these high-frequency fluctuations is the random noise imposed on the regular fluctuations.

Once this random noise is eliminated (by setting the variance of $u_{t}$ and $v_{t}$ to zero) the dependence of the phase difference on the lower bound is only present in the middle of the sample where the phase shift changes (here for a range of roughly 100 observations from 250 to 350) but otherwise vanishes. In this region around 300 the wavelet coherence in Fig. 9 is much lower than before and after. This is also visible from the black lines indicating that coherence is not significantly different from zero in this region. Therefore, it is common practice in wavelet analysis to put emphasis on the interpretation of the wavelet phase difference only when coherence is substantial.

## Footnotes

1 Frequency domain analyses of leading indicators are provided by Entorf (1993) as well as Rua and Nunes (2005)

2 The stretching and compressing of the wavelet by *s* leads to a flexible window length depending on the scale (and therefore the frequency) in contrast to a constant window length which is the basis of windowed Fourier analysis.

3 On may raise concerns about this procedure since it may lead to size distortions as stressed by a reviewer. The significance regions of the plot are less relevant than is the magnitude of the coherence. We interpret the significance regions merely as a suggestive indication of regions with high coherence.

4 With periodic time series data the fast Fourier transformation used for computation is usually padded by zeros at both edges of the time series. This removes detrimental effects at the edges but induces a discontinuity and leads to less precise estimates of the wavelet power spectrum at the edges. The resulting conical region is called the cone of influence (COI) and is depicted in light shades in the figures below. Its conical shape originates from the fact that more padding is necessary for larger scales (lower frequencies) for a given time series length. Torrence and Compo (1998) provide details of the computation.

5 See Döpke et al. (2019) for survey evidence that even professional forecasters tend to prefer well-known and simple forecasting methods over more recently developed sophisticated methods.

6 Seasonally adjusted time series are also frequently used in the literature. Using the unadjusted counterparts of the series does not change the principal findings reported below.

7 This time series should be stationary by construction as argued by Lehmann (2020). A time series plot of the levels shows some features of unit root dynamics. The ERS test (Elliott et al. 1996) rejects its unit root null for both levels and annual differences. The KPSS test (Kwiatkowski et al. 1992) for the stationary null rejects for the levels but not for the annual differences. Because of this outcome and because the annual differences are a meaningful indicator we opted for annual differencing.

8 This transformation reduces very short-run fluctuations which would be emphasized by taking the difference to the previous month and would mask the business cycle fluctuations we want to focus on. It is likewise employed by Caraiani (2012) for quarterly data.

9 See Baxter and King (1999), referring to earlier research.

10 Recall that we are analyzing changes so that the average spectra naturally deviate from the “typical spectral shape of an economic variable” according to Granger (1966).

11 Caraiani (2012), Gallegati (2014b) chose a more narrow band of 4–8 years. We find that the common band ob business cycle frequencies of 2–8 years is here suitable to stabilize the lead-lag relationships.

12 The general result of stock market returns leading economic activity is also established by Gallegati (2014a) using discrete wavelet analysis.
